# Supplementary material for: Hot spot prediction in protein-protein interactions by an ensemble system
Source: BMC Syst Biol. 2018 Dec 31;12(Suppl 9):132. doi: 10.1186/s12918-018-0665-8 (PMC6311905; doi:10.1186/s12918-018-0665-8)
Supplement: Supplementary file 2 — All relevant MATLAB code. A simple Matlab implement of our predictor. (ZIP 19,742 kb) [file 12918_2018_665_MOESM2_ESM.zip › Additional file 2/Read ASA/ASA website/BID/1FE8A.html]

 NetSurfP - Protein Surface Accessibility and Secondary Structure Predictions

  

|  |  |  |
| --- | --- | --- |
|  |  | NetSurfP - Protein Surface Accessibility and Secondary Structure Predictions  Technical University of Denmark |

  


---

```
# For publication of results, please cite:
# A generic method for assignment of reliability scores applied to solvent accessibility predictions.
# Bent Petersen, Thomas Nordahl Petersen, Pernille Andersen, Morten Nielsen and Claus Lundegaard
# BMC Structural Biology 2009, 9:51 doi:10.1186/1472-6807-9-51
#
# Column 1: Class assignment - B for buried or E for Exposed - Threshold: 25% exposure, but not based on RSA
# Column 2: Amino acid
# Column 3: Sequence name
# Column 4: Amino acid number
# Column 5: Relative Surface Accessibility - RSA
# Column 6: Absolute Surface Accessibility
# Column 7: Z-fit score for RSA prediction
# Column 8: Probability for Alpha-Helix
# Column 9: Probability for Beta-strand
# Column 10: Probability for Coil
E G  1FE8A                  1    0.893  70.279  -2.309   0.003   0.003   0.994
E S  1FE8A                  2    0.705  82.673   0.485   0.018   0.088   0.893
E H  1FE8A                  3    0.694 126.293   1.065   0.020   0.205   0.775
E M  1FE8A                  4    0.317  63.412  -0.545   0.019   0.141   0.840
E A  1FE8A                  5    0.579  63.817   0.800   0.018   0.088   0.893
E P  1FE8A                  6    0.652  92.590   0.560   0.018   0.088   0.893
E D  1FE8A                  7    0.545  78.477   0.571   0.019   0.141   0.840
E C  1FE8A                  8    0.337  47.329   0.113   0.018   0.088   0.893
E S  1FE8A                  9    0.655  76.778   0.189   0.018   0.088   0.893
E Q  1FE8A                 10    0.519  92.658   0.535   0.018   0.047   0.935
E P  1FE8A                 11    0.346  49.126   0.301   0.005   0.045   0.951
B L  1FE8A                 12    0.066  12.085   0.762   0.004   0.420   0.576
B D  1FE8A                 13    0.107  15.476   0.252   0.002   0.816   0.182
B V  1FE8A                 14    0.022   3.366   1.390   0.001   0.959   0.040
B I  1FE8A                 15    0.022   4.089   1.187   0.000   0.983   0.017
B L  1FE8A                 16    0.017   3.076   1.800   0.000   0.983   0.017
B L  1FE8A                 17    0.015   2.765   1.666   0.001   0.959   0.040
B L  1FE8A                 18    0.026   4.797   1.026   0.001   0.900   0.099
B D  1FE8A                 19    0.051   7.349   0.471   0.003   0.718   0.279
B G  1FE8A                 20    0.166  13.041  -0.289   0.019   0.141   0.840
B S  1FE8A                 21    0.078   9.188  -0.814   0.018   0.047   0.935
E S  1FE8A                 22    0.377  44.208  -0.849   0.018   0.047   0.935
B S  1FE8A                 23    0.236  27.624  -1.800   0.018   0.019   0.964
B F  1FE8A                 24    0.155  31.129  -0.718   0.018   0.019   0.964
E P  1FE8A                 25    0.438  62.138   0.266   0.018   0.019   0.964
E A  1FE8A                 26    0.583  64.247  -0.164   0.502   0.002   0.495
E S  1FE8A                 27    0.675  79.075   0.155   0.406   0.004   0.590
E Y  1FE8A                 28    0.352  75.308   1.026   0.321   0.003   0.675
B F  1FE8A                 29    0.053  10.617   0.714   0.923   0.002   0.076
E D  1FE8A                 30    0.565  81.474   1.935   0.970   0.001   0.030
E E  1FE8A                 31    0.342  59.712   2.134   0.970   0.001   0.030
B M  1FE8A                 32    0.020   4.082   1.198   0.970   0.001   0.030
B K  1FE8A                 33    0.154  31.678   1.326   0.970   0.001   0.030
E S  1FE8A                 34    0.523  61.284   1.920   0.988   0.000   0.012
B F  1FE8A                 35    0.042   8.369   0.329   0.988   0.000   0.012
B A  1FE8A                 36    0.015   1.620   1.388   0.988   0.000   0.012
E K  1FE8A                 37    0.289  59.530   1.732   0.988   0.000   0.012
E A  1FE8A                 38    0.351  38.724   2.060   0.988   0.000   0.012
B F  1FE8A                 39    0.032   6.362   0.342   0.970   0.001   0.030
B I  1FE8A                 40    0.045   8.233   0.207   0.970   0.001   0.030
E S  1FE8A                 41    0.511  59.842   1.386   0.923   0.002   0.076
E K  1FE8A                 42    0.324  66.667   1.126   0.858   0.002   0.139
B A  1FE8A                 43    0.042   4.628  -0.289   0.694   0.003   0.303
E N  1FE8A                 44    0.473  69.306   0.147   0.502   0.002   0.495
E I  1FE8A                 45    0.521  96.348  -1.214   0.246   0.004   0.750
E G  1FE8A                 46    0.379  29.843  -1.079   0.053   0.005   0.942
E P  1FE8A                 47    0.525  74.455  -0.551   0.018   0.019   0.964
E R  1FE8A                 48    0.640 146.514  -0.397   0.018   0.019   0.964
E L  1FE8A                 49    0.291  53.374   0.102   0.005   0.015   0.979
B T  1FE8A                 50    0.047   6.519  -0.211   0.004   0.197   0.799
B Q  1FE8A                 51    0.094  16.806   0.788   0.002   0.816   0.182
B V  1FE8A                 52    0.022   3.320   1.074   0.001   0.959   0.040
B S  1FE8A                 53    0.019   2.262   0.976   0.001   0.959   0.040
B V  1FE8A                 54    0.016   2.428   1.514   0.001   0.959   0.040
B L  1FE8A                 55    0.022   4.083   1.020   0.001   0.959   0.040
B Q  1FE8A                 56    0.079  14.163   0.635   0.001   0.959   0.040
B Y  1FE8A                 57    0.056  12.031   0.293   0.001   0.900   0.099
B G  1FE8A                 58    0.151  11.899  -0.764   0.004   0.085   0.910
E S  1FE8A                 59    0.472  55.342  -0.835   0.005   0.015   0.979
E I  1FE8A                 60    0.465  86.006   0.473   0.005   0.262   0.733
B T  1FE8A                 61    0.079  10.916  -0.364   0.004   0.514   0.481
E T  1FE8A                 62    0.304  42.165   0.623   0.018   0.846   0.136
B I  1FE8A                 63    0.231  42.735   0.235   0.001   0.959   0.040
B D  1FE8A                 64    0.251  36.140   0.206   0.001   0.900   0.099
B V  1FE8A                 65    0.077  11.804   0.769   0.003   0.718   0.279
E P  1FE8A                 66    0.384  54.532   0.295   0.004   0.514   0.481
B W  1FE8A                 67    0.093  22.294  -0.539   0.004   0.138   0.858
E N  1FE8A                 68    0.368  53.802  -0.299   0.005   0.045   0.951
E V  1FE8A                 69    0.509  78.172  -0.063   0.053   0.043   0.903
E V  1FE8A                 70    0.348  53.549  -0.748   0.430   0.016   0.555
E P  1FE8A                 71    0.491  69.701  -0.817   0.339   0.016   0.645
E E  1FE8A                 72    0.360  62.857  -0.353   0.181   0.016   0.803
E K  1FE8A                 73    0.252  51.919   0.634   0.970   0.001   0.030
E A  1FE8A                 74    0.568  62.572   0.486   0.970   0.001   0.030
E H  1FE8A                 75    0.387  70.323   1.632   0.970   0.001   0.030
B L  1FE8A                 76    0.041   7.544   0.009   0.970   0.001   0.030
E L  1FE8A                 77    0.273  50.041   0.910   0.970   0.001   0.030
E S  1FE8A                 78    0.467  54.709   1.554   0.970   0.001   0.030
B L  1FE8A                 79    0.128  23.473   0.401   0.970   0.001   0.030
B V  1FE8A                 80    0.048   7.331  -0.658   0.923   0.002   0.076
E D  1FE8A                 81    0.558  80.451   0.963   0.782   0.003   0.216
E V  1FE8A                 82    0.562  86.426   1.254   0.600   0.003   0.397
B M  1FE8A                 83    0.077  15.308   0.028   0.430   0.016   0.555
E Q  1FE8A                 84    0.498  88.889   0.618   0.339   0.016   0.645
E R  1FE8A                 85    0.389  89.012  -0.756   0.339   0.016   0.645
E E  1FE8A                 86    0.516  90.128  -0.968   0.176   0.004   0.820
E G  1FE8A                 87    0.555  43.702  -2.746   0.053   0.005   0.942
E G  1FE8A                 88    0.426  33.495  -1.666   0.053   0.005   0.942
B P  1FE8A                 89    0.244  34.609  -1.568   0.058   0.017   0.925
B S  1FE8A                 90    0.058   6.798  -1.976   0.113   0.043   0.844
B Q  1FE8A                 91    0.158  28.165  -0.105   0.430   0.016   0.555
B I  1FE8A                 92    0.044   8.140  -0.655   0.938   0.007   0.055
B G  1FE8A                 93    0.116   9.098  -0.910   0.970   0.001   0.030
E D  1FE8A                 94    0.287  41.342   1.005   0.970   0.001   0.030
B A  1FE8A                 95    0.019   2.083   0.303   0.970   0.001   0.030
B L  1FE8A                 96    0.028   5.108   0.540   0.988   0.000   0.012
E G  1FE8A                 97    0.296  23.327   1.457   0.988   0.000   0.012
B F  1FE8A                 98    0.217  43.572   1.876   0.970   0.001   0.030
B A  1FE8A                 99    0.014   1.499   1.210   0.988   0.000   0.012
B V  1FE8A                100    0.107  16.477   0.592   0.970   0.001   0.030
E R  1FE8A                101    0.547 125.332   1.677   0.970   0.001   0.030
B Y  1FE8A                102    0.201  42.911   0.943   0.970   0.001   0.030
B L  1FE8A                103    0.033   5.969  -0.342   0.923   0.002   0.076
E T  1FE8A                104    0.317  43.940   0.276   0.858   0.002   0.139
E S  1FE8A                105    0.562  65.913   0.414   0.782   0.003   0.216
E E  1FE8A                106    0.402  70.264  -0.865   0.600   0.003   0.397
E M  1FE8A                107    0.555 111.056  -1.316   0.406   0.004   0.590
E H  1FE8A                108    0.569 103.537  -1.127   0.246   0.004   0.750
E G  1FE8A                109    0.560  44.041  -1.829   0.109   0.005   0.886
E A  1FE8A                110    0.444  48.885  -2.031   0.053   0.005   0.942
E R  1FE8A                111    0.331  75.753  -0.816   0.058   0.017   0.925
E P  1FE8A                112    0.658  93.342  -1.051   0.058   0.017   0.925
E G  1FE8A                113    0.602  47.385  -0.984   0.018   0.019   0.964
B A  1FE8A                114    0.176  19.428  -0.288   0.005   0.045   0.951
E S  1FE8A                115    0.310  36.344   0.566   0.005   0.262   0.733
B K  1FE8A                116    0.178  36.532   1.065   0.003   0.718   0.279
B A  1FE8A                117    0.042   4.595   0.950   0.001   0.959   0.040
B V  1FE8A                118    0.013   1.967   1.754   0.001   0.959   0.040
B V  1FE8A                119    0.015   2.229   1.705   0.000   0.983   0.017
B I  1FE8A                120    0.016   2.923   1.655   0.001   0.959   0.040
B L  1FE8A                121    0.021   3.863   1.244   0.001   0.959   0.040
B V  1FE8A                122    0.030   4.550   0.121   0.003   0.718   0.279
B T  1FE8A                123    0.147  20.389   0.459   0.004   0.138   0.858
B D  1FE8A                124    0.202  29.108  -0.862   0.005   0.015   0.979
E V  1FE8A                125    0.467  71.747   0.217   0.005   0.015   0.979
B S  1FE8A                126    0.256  29.968  -0.775   0.018   0.019   0.964
E V  1FE8A                127    0.493  75.805  -1.551   0.053   0.005   0.942
E D  1FE8A                128    0.537  77.425  -1.522   0.109   0.005   0.886
E S  1FE8A                129    0.427  50.068   0.516   0.109   0.005   0.886
B V  1FE8A                130    0.050   7.624  -0.335   0.858   0.002   0.139
E D  1FE8A                131    0.421  60.637   1.150   0.858   0.002   0.139
E A  1FE8A                132    0.533  58.792   1.644   0.923   0.002   0.076
B A  1FE8A                133    0.041   4.562  -0.045   0.970   0.001   0.030
B A  1FE8A                134    0.024   2.634   0.238   0.970   0.001   0.030
E D  1FE8A                135    0.527  75.926   1.754   0.970   0.001   0.030
E A  1FE8A                136    0.360  39.650   1.805   0.970   0.001   0.030
B A  1FE8A                137    0.034   3.747  -0.954   0.923   0.002   0.076
E R  1FE8A                138    0.443 101.355   0.891   0.858   0.002   0.139
E S  1FE8A                139    0.805  94.311   0.793   0.600   0.003   0.397
E N  1FE8A                140    0.486  71.209   0.863   0.053   0.005   0.942
E R  1FE8A                141    0.689 157.827   0.664   0.005   0.015   0.979
B V  1FE8A                142    0.030   4.657   0.550   0.015   0.102   0.884
E T  1FE8A                143    0.299  41.402   2.012   0.002   0.816   0.182
B V  1FE8A                144    0.030   4.688   0.722   0.000   0.983   0.017
B F  1FE8A                145    0.044   8.791   1.148   0.000   0.983   0.017
B P  1FE8A                146    0.018   2.583   0.998   0.000   0.983   0.017
B I  1FE8A                147    0.030   5.550   0.633   0.001   0.959   0.040
B G  1FE8A                148    0.029   2.290  -1.420   0.001   0.900   0.099
B I  1FE8A                149    0.055  10.156   0.343   0.004   0.514   0.481
B G  1FE8A                150    0.099   7.831  -0.962   0.005   0.045   0.951
E D  1FE8A                151    0.569  81.935   0.013   0.005   0.015   0.979
E R  1FE8A                152    0.594 136.095  -0.928   0.018   0.019   0.964
B Y  1FE8A                153    0.189  40.325   0.314   0.016   0.005   0.979
E D  1FE8A                154    0.387  55.781   0.568   0.016   0.005   0.979
E A  1FE8A                155    0.376  41.479   1.512   0.970   0.001   0.030
E A  1FE8A                156    0.634  69.834   1.492   0.970   0.001   0.030
B Q  1FE8A                157    0.180  32.148   1.575   0.970   0.001   0.030
B L  1FE8A                158    0.019   3.442   1.258   0.970   0.001   0.030
E R  1FE8A                159    0.402  92.058   2.265   0.970   0.001   0.030
E I  1FE8A                160    0.484  89.485   2.090   0.970   0.001   0.030
B L  1FE8A                161    0.026   4.852   0.671   0.923   0.002   0.076
B A  1FE8A                162    0.034   3.769  -1.431   0.782   0.003   0.216
E G  1FE8A                163    0.678  53.327   0.434   0.406   0.004   0.590
E P  1FE8A                164    0.374  53.056   0.804   0.176   0.004   0.820
E A  1FE8A                165    0.546  60.158  -1.382   0.053   0.005   0.942
E G  1FE8A                166    0.642  50.510  -1.820   0.003   0.003   0.994
E D  1FE8A                167    0.470  67.684  -1.392   0.005   0.015   0.979
E S  1FE8A                168    0.520  60.979   0.118   0.004   0.138   0.858
B N  1FE8A                169    0.132  19.354   0.159   0.003   0.718   0.279
B V  1FE8A                170    0.146  22.486   0.153   0.001   0.900   0.099
B V  1FE8A                171    0.073  11.174   0.394   0.001   0.900   0.099
E K  1FE8A                172    0.256  52.659   0.374   0.001   0.900   0.099
B L  1FE8A                173    0.056  10.309  -0.417   0.004   0.616   0.381
E Q  1FE8A                174    0.468  83.674   0.058   0.004   0.197   0.799
E R  1FE8A                175    0.471 107.859   0.162   0.018   0.088   0.893
E I  1FE8A                176    0.277  51.282  -0.360   0.257   0.016   0.727
E E  1FE8A                177    0.703 122.779  -0.441   0.176   0.004   0.820
E D  1FE8A                178    0.452  65.162   0.630   0.176   0.004   0.820
B L  1FE8A                179    0.095  17.413   0.366   0.782   0.003   0.216
E P  1FE8A                180    0.645  91.455   1.045   0.858   0.002   0.139
E T  1FE8A                181    0.416  57.727   1.394   0.923   0.002   0.076
B M  1FE8A                182    0.032   6.383   0.225   0.970   0.001   0.030
E V  1FE8A                183    0.342  52.612   1.318   0.970   0.001   0.030
E T  1FE8A                184    0.611  84.746   1.923   0.970   0.001   0.030
B L  1FE8A                185    0.098  17.980   0.512   0.970   0.001   0.030
B G  1FE8A                186    0.088   6.933   0.287   0.970   0.001   0.030
E N  1FE8A                187    0.552  80.784   1.475   0.970   0.001   0.030
E S  1FE8A                188    0.493  57.780   2.213   0.970   0.001   0.030
B F  1FE8A                189    0.046   9.252   0.591   0.970   0.001   0.030
B L  1FE8A                190    0.194  35.576   0.738   0.970   0.001   0.030
E H  1FE8A                191    0.601 109.395   1.586   0.923   0.002   0.076
E K  1FE8A                192    0.440  90.487   1.868   0.858   0.002   0.139
B L  1FE8A                193    0.172  31.566  -0.223   0.782   0.003   0.216
B C  1FE8A                194    0.161  22.590  -1.032   0.522   0.016   0.462
E S  1FE8A                195    0.657  76.942   1.290   0.181   0.016   0.803
E G  1FE8A                196    0.787  61.921  -1.552   0.003   0.003   0.994

Explain the output.  Go back.
```

---
